# Loss of functional diversity and network modularity in introduced plant–fungal symbioses

**DOI:** 10.1093/aobpla/plw084

**Published:** 2017-02-05

**Authors:** Ian A. Dickie, Jerry A. Cooper, Jennifer L. Bufford, Philip E. Hulme, Scott T. Bates

**Affiliations:** 1 Bio-Protection Research Centre, Lincoln University, Box 85084, Lincoln 7647, New Zealand; 2 Landcare Research, PO Box 69040, Lincoln 7640, New Zealand; 3 Department of Plant Pathology, University of Minnesota, Saint Paul, MN 55108, USA

**Keywords:** Bipartite interaction networks, ectomycorrhizal symbiosis, foraging strategies, functional traits, invasion ecology, network modularity, second-genome, soil ecology

## Abstract

The introduction of alien plants into a new range can result in the loss of co-evolved symbiotic organisms, such as mycorrhizal fungi, that are essential for normal plant physiological functions. Prior studies of mycorrhizal associations in alien plants have tended to focus on individual plant species on a case-by-case basis. This approach limits broad scale understanding of functional shifts and changes in interaction network structure that may occur following introduction. Here we use two extensive datasets of plant–fungal interactions derived from fungal sporocarp observations and recorded plant hosts in two island archipelago nations: New Zealand (NZ) and the United Kingdom (UK). We found that the NZ dataset shows a lower functional diversity of fungal hyphal foraging strategies in mycorrhiza of alien when compared with native trees. Across species this resulted in fungal foraging strategies associated with alien trees being much more variable in functional composition compared with native trees, which had a strikingly similar functional composition. The UK data showed no functional difference in fungal associates of alien and native plant genera. Notwithstanding this, both the NZ and UK data showed a substantial difference in interaction network structure of alien trees compared with native trees. In both cases, fungal associates of native trees showed strong modularity, while fungal associates of alien trees generally integrated into a single large module. The results suggest a lower functional diversity (in one dataset) and a simplification of network structure (in both) as a result of introduction, potentially driven by either limited symbiont co-introductions or disruption of habitat as a driver of specificity due to nursery conditions, planting, or plant edaphic-niche expansion. Recognizing these shifts in function and network structure has important implications for plant invasions and facilitation of secondary invasions via shared mutualist populations.

## Teaser

Native and alien trees associate with a wide range of beneficial fungi, but the few studies of these interactions tend to focus only on a few plant species or locations at a time. Using extensive databases collected by mycologists in the United Kingdom and New Zealand, we show that, in the latter region, fungi on alien trees are less functionally diverse than those associated with natives. In both New Zealand and the United Kingdom, however, the structure of the interaction network is simplified and "nested". This suggests that beneficial fungi hosted by alien trees may help facilitate further tree invasion.

## Introduction

When plants are introduced to a new landmass, they are often transported with a greatly reduced number of associated organisms, including many symbiotic mutualists (Richardson *et al.* 2000; Nuñez and Dickie 2014; Klock *et al.* 2016; Burgess *et al.* 2016). In principle, this could represent a loss or change of physiological functions associated with a plant, as most plants rely on symbionts for normal nutrient uptake (Brundrett 2009). One well-studied example is ectomycorrhizal fungi, which associate with many widely introduced tree species (Richardson *et al.* 1994; Tedersoo *et al.* 2007; Dickie *et al.* 2010). The initial lack of compatible fungi and their functional capabilities helps explain the failure of some early efforts to introduce alien trees and continues to limit certain tree invasions (Nuñez *et al.* 2009; Dickie *et al.* 2010). Nonetheless, the successful invasion of *Pinus* associating with a highly simplified fungal community suggests that even where there is a major reduction in mycorrhizal associates, trees may not be limited by a loss of symbiont diversity (Hynson *et al.* 2013; Hayward *et al.* 2015*b*). Furthermore, in some cases alien trees may form novel associations with fungal associates of native flora (Tedersoo *et al.* 2007; Bahram *et al.* 2013; Moeller *et al.* 2015), or re-establish symbioses with cosmopolitan species (Dickie *et al.* 2010).

Our understanding of ectomycorrhizal symbioses of alien species is primarily based on studies of *Pinus*, which frequently co-invades with the host-specific, animal-dispersed fungal genus *Suillus* (Nuñez *et al.* 2013; Nuñez and Dickie, 2014; Hayward *et al.* 2015*b*; Wood *et al.* 2015). The few studies of other alien ectomycorrhizal trees, including *Eucalyptus* (Diez 2005)*, Pseudotsuga* (Moeller *et al.* 2015), *Salix* and *Alnus* (Bogar *et al.* 2015) suggest that co-invasion with a reduced fungal community from the native range is widespread, although the identity of co-invading fungi may vary with alien tree identity.

The identity of fungal symbionts has substantial significance for plant phenotypes, as fungi differ greatly in their hyphal foraging strategies (Agerer 2001), vertical niche preferences in soil (Dickie *et al.* 2002), ability to use organic nutrients (Abuzinadah and Read 1986; Lilleskov *et al.* 2002), and other key traits that link directly to plant nutrient uptake (Hobbie and Agerer 2010; Tedersoo *et al.* 2012; Hobbie *et al.* 2014; Koide *et al.* 2014). We consider the hyphal foraging strategies of fungi associated with a plant to represent the functional traits of that plant species. Having a high functional diversity of different hyphal foraging strategies may allow a plant population greater access to different nutrient sources, and greater adaptability to changing environments. Indeed, the genetic traits of ectomycorrhizal fungi could be considered as the ‘second-genome’ of plants (Zenni *et al.* 2016*a*), in the same sense as the term has been applied to endosymbionts of animals (Shah *et al.* 2016). As such, a complete understanding of evolutionary dynamics in tree invasions requires understanding of not just genomic traits (Zenni *et al.* 2016*b*) and phenotypic plasticity (Zimmermann *et al.* 2016), but also traits of associated symbionts.

An ectomycorrhizal fungal species can associate with multiple, potentially distantly related, plant species (Simard and Durall 2004). This means that the presence of one plant species may support a population of fungi that serves as inoculum, facilitating the establishment of a second plant species. Shared fungal symbiont species have been observed between co-occurring alien *Alnus* and *Salix* (Bogar *et al.* 2015), and between alien trees, *Pinus* and *Pseudotsuga*, and native trees in the Nothofagaceae (Dickie *et al.* 2010; Moeller *et al.* 2015), although the shared symbionts are generally only a small portion of total associations. Shared fungal symbiont species have also been observed between native trees and alien trees in plantations of *Eucalyptus* (Tedersoo *et al.* 2007) and *Pinus* (Bahram *et al.* 2013). Further shared symbionts can occur where novel associations form between alien trees and alien fungi from different origins, such as Australian *Eucalyptus* associating with northern-hemisphere *Amanita muscaria* in New Zealand (Nuñez and Dickie, 2014).

The interactions of alien trees through shared mutualist species can be analysed through network theory (Nuismer *et al.* 2013; Roy Bolduc *et al.* 2016; Le Roux *et al.* 2016). Network theory allows analysis of emergent properties of interactions between multiple partners, including nestedness (the degree to which generalists associate with specialists, and *vice versa*), and the identification of modules within the network (species that tend to share partners more than expected by chance), and quantification of modularity (the density of links within as opposed to between modules). In mycorrhizal ecology ‘mycorrhizal networks’ or ‘common mycorrhizal networks’ have been used to describe physical linkages between individual plants through a single fungal mycelium (Simard and Durall 2004; Horton 2015). This is not what we mean by ‘interaction network.’ Here we refer to ‘interaction network’ as the potential for plants to share fungal symbionts at the level of populations or species. This usage is common in other symbioses, for example in pollination biology (Santos *et al.* 2012; Nuismer *et al.* 2013; Traveset *et al.* 2013). Unlike the specific case of mycorrhizal networks, interaction networks do not necessarily imply a link through a single individual fungus, any transfer of resources, or any physiological effects above-and-beyond changes in populations of fungi. Nonetheless, these changes in fungal populations can be critical, as increased nestedness of mutualistic networks is believed to increase network stability (Thebault and Fontaine 2010), while modularity may make networks less stable and prone to alternative stable states. Previous studies of ectomycorrhizal interaction networks suggest a nonnested or antinested pattern, with either no or less nestedness than would be expected by random chance, respectively (Bahram *et al.* 2014; Roy Bolduc *et al.* 2016). Antinested networks can arise from strong selection for fungus-host specificity (Nuismer *et al.* 2013). While alien ectomycorrhizal mutualistic networks have not previously been studied, data from pollinator networks suggests that aliens can be more generalist in their associations than natives, reducing network modularity and making networks more nested (Santos *et al.* 2012; Traveset *et al.* 2013).

On the basis of the above background, we set out to test the hypotheses:

H1 – alien plants will have a reduced functional diversity of ectomycorrhizal symbionts compared to native plants.

H2 – loss of specialist symbionts on alien plant species will result in a less modular structure of plant–fungal interactions among aliens compared to native plants (i.e. more sharing of fungal symbiont species across plant species).

We used data from two island nations: New Zealand and the UK. Both nations have a large number of introduced alien tree species, native ectomycorrhizal trees and relatively well-described fungal communities.

## Methods

Empirical testing of hypotheses about fungal community structure across large numbers of plant species is challenging due to the below-ground nature of the interaction and the cost of molecular approaches necessary to identify fungi on plant roots. However, many ectomycorrhizal fungi produce highly visible fruiting structures, such as mushrooms (also known as sporocarps or carpophores), that reveal the symbiotic interaction between the fungal mycelium and plant roots below ground. Furthermore, many active amateur as well professional mycologists record the presence of these mushrooms and often also record the presence of a putative host plant.

Data on putative plant–fungal associations in New Zealand was obtained from the New Zealand Fungal and Plant Disease Collection (PDD) database of Landcare Research [http://www.landcareresearch.co.nz (1 November 2016)]. Similar data from the UK was obtained from the Fungal Records Database of Britain and Ireland, maintained by the British Mycological Society [http://www.fieldmycology.net/ (1 November 2016)]. The databases comprise a combination of professional and amateur observations of fungi including coordinated collections from annual ‘fungal foray’ events, ad hoc collections, and literature records. In New Zealand, the Fungal Network of New Zealand has been very active, and encourages uniform data standards by providing datasheets for all collections during annual foray events.

One of the limitations in working with data collected by amateur field mycologists is that the quality of plant identifications can be lower than the quality of fungal identifications. In particular, many collections were labelled with a plant genus, but not a species. Furthermore, some of the plants recorded as ‘hosts’ of ectomycorrhizal fungi are not known to form ectomycorrhizal associations (Brundrett 2009) based on a phylogenetic understanding of mycorrhizal status (Koele *et al.* 2012). We therefore filtered tree genera to retain only those known to be ectomycorrhizal (Brundrett 2009; Koele *et al.* 2012) and analysed data at the plant genus level. The genus *Nothofagus* has recently been split into four genera, with all native New Zealand *Nothofagus* now classified as either *Lophozonia* (*L. menziesii*) or *Fuscospora* (*F. solandri*, *F. fusca*, and *F. truncata*), while two aliens in our data, *N. antarctica* and *N. nitida*, remain in the genus *Nothofagus* (Heenan and Smissen 2013). We therefore were only able to retain *Nothofagus* records where the plant species was recorded, despite analysing our data at the plant genus level. Fortunately, the native Nothofagaceae are dominant tree species in NZ, so *Lophozonia* and *Fuscospora* remained our two best-represented plant genera despite this restriction. For the UK data, we retained the native *Pinus sylvestris* as distinct from alien pine species. Out of necessity, native status of plants was designated at the level of national boundaries (e.g. *Pinus sylvestris* was considered native to the whole of the UK, despite only occurring naturally in Scotland). For both datasets we retained fungi identified only to genus, but used species level identifications where available. To test whether this might bias the results we also analysed the data excluding genus-only identifications, and found no qualitative changes in results.

### Ectomycorrhizal status and exploration type analysis

Ectomycorrhizal status of fungi was assigned based on Comandini *et al.* (2012) that builds on earlier works (Rinaldi *et al.* 2008; Tedersoo *et al.* 2010). Similarly, hyphal exploration types were assigned to ectomycorrhizal fungi on the basis of a comprehensive review (Tedersoo and Smith 2013). Where a species was listed in Comandini *et al.* (2012) but not in Tedersoo and Smith (2013), we evaluated it on a case-by-case basis. Based on our assessment of the conflicting evidence we classified *Chalciporus*, *Chloridium*, *Gyromitra*, *Lachnum*, *Leptodontidium, Leucopaxillus* (Vizzini 2012), *Paurocotylis* and *Phialocephala* as non-ectomycorrhizal fungi.

While ectomycorrhizal fungi vary in many fundamental traits (Koide *et al.* 2014), fungal exploration types are one of the few traits that have been extensively studied across a wide range of species (Agerer 2001; Hobbie and Agerer 2010). Exploration types were initially catalogued as morphological traits for microscopic identification of fungi (Agerer 1997), with categorical classifications based on the abundance and length of hyphae. These trait categories have subsequently been shown to be closely correlated with nutrient uptake capabilities (Hobbie and Agerer 2010). Exploration types with relatively low biomass of hyphae (contact, short and medium-smooth types) are hydrophilic, and may be adapted for uptake of labile nitrogen (amino acids, ${\text{NH}}_{4}^{\text{+}}$, ${\text{NO}}_{3}^{-}$). Exploration types with higher hyphal biomass and longer hyphae (medium-fringe, medium-matt and long-distance) are hydrophobic and may be adapted for more spatially dispersed organic N sources, such as proteins (Hobbie and Agerer 2010). Fungal exploration types have been catalogued at the fungal genus level in a comprehensive review by Tedersoo and Smith (2013), although some variation within genus does occur.

We summed all ectomycorrhizal sporocarp observations by hyphal foraging type within ectomycorrhizal tree genera. Tree genera with fewer than 8 sporocarp collections in a region were excluded. Functional diversity was calculated as Shannon diversity (*H*′) and compositional differences visualized using principle components analysis. In order to test and account for any bias in *H*′ for smaller sample size, we randomized the community matrix, maintaining row and column totals constant, using the function permatswap in the R package vegan (Oksanen *et al.* 2010) with a burn-in of 99 swaps discarded, and 9999 swaps analysed. We tested differences in dispersion of functional associations between native and alien trees using the function betadisper, based on Mahalanobis distances.

### Network analysis

Analysis of networks was based on fungal species recorded as associating with different plant genera. As our interest was in community structure of interaction linkages between plant species, we followed Fortuna *et al.* (2010) in reducing the bi-partite interaction network (in which plants and fungi comprise two sets of nodes) to a unipartite projection (where fungi represent links between plant nodes). So, for example, if fungal species FS1 occurs on plant genus PG1 and on plant genus PG2, we consider this to represent a potential link between PG1 and PG2. The unipartite projection provided a clear reflection of our hypothesis, which was fundamentally about the sharing of fungal symbionts across plant species. To create the unipartite projection, fungal links were weighted on the basis of the smaller *n*. For example, if a fungal species FS1 occurred 15 times on one plant genus PG1 and 3 times on plant genus PG2, the link between PG1 and PG2 would have a weight of 3. We were specifically interested in maintaining self–self linkages, such that host-specific fungi would be retained in the analysis. We therefore included each fungus as a self–self link with a weight of the number of occurrences (e.g. FS1 would be represented as a PG1 to PG1 link of Weight 15, a PG2 to PG2 link of Weight 3, following the above example).

There are many methods for the detection of modules (or communities) within interaction networks, but with varying levels of support (Lancichinetti and Fortunato 2009). After reviewing these, along with ease of implementation, we used the cluster_louvain method in the igraph package of R, which is based on Blondel *et al.* (2008) and supported by Lancichinetti and Fortunato (2009) on the basis of comparison against benchmarks and random graphs. We also explored other techniques for detecting modules (e.g. spinglass, walktrap, leading eigenvector methods) and found that, in general, they varied in the number of modules (from a minimum of 1 to a maximum of the number of plant genera in the dataset) but rarely produced conflicting memberships in modules (not shown).

## Results

### Data set

For the NZ dataset, we identified 4000 sporocarp records of 637 ectomycorrhizal fungal taxa associated with 19 tree genera (Table 1; **See Supporting Information—Table S1**). For the UK dataset we identified 164 901 sporocarp records of 2304 ectomycorrhizal fungal taxa associated with 19 tree genera (Table 1; **See Supporting Information—Table S2**). Diversity of fungi (*H*′) by tree genus was higher on native than alien trees (Table 1), but the difference was only significant in New Zealand (*t*-test, *t*_16.98_*_ _= −*8.959, *P* < 0.001), not the UK (*t*-test, *t*_14.86__* *_ =  *−*1.7932, *P*  =  0.09).

**Table 1.** Summary of data on plant–fungal interactions and diversity of fungi by region and origin of host plants.

Region	Plant genera	Total fungal observations	Number of fungal species	Fungal diversity (*H*′)^1^^,^^2^
NZ	Native (*n* = 4)	3114	427	4.67 ± 0.12***
	Exotic (*n* = 15)	812	152	2.10 ± 0.26
UK	Native (*n* = 10)	153 580	1603	4.62 ± 0.22
	Exotic (*n* = 9)	11 321	701	3.94 ± 0.31

^1^ Shannon diversity of fungi associated with each tree genus, mean and SE.

^2^ Stars indicate significant differences within region (*** < 0.001).

### Functional diversity

**Figure 1:**
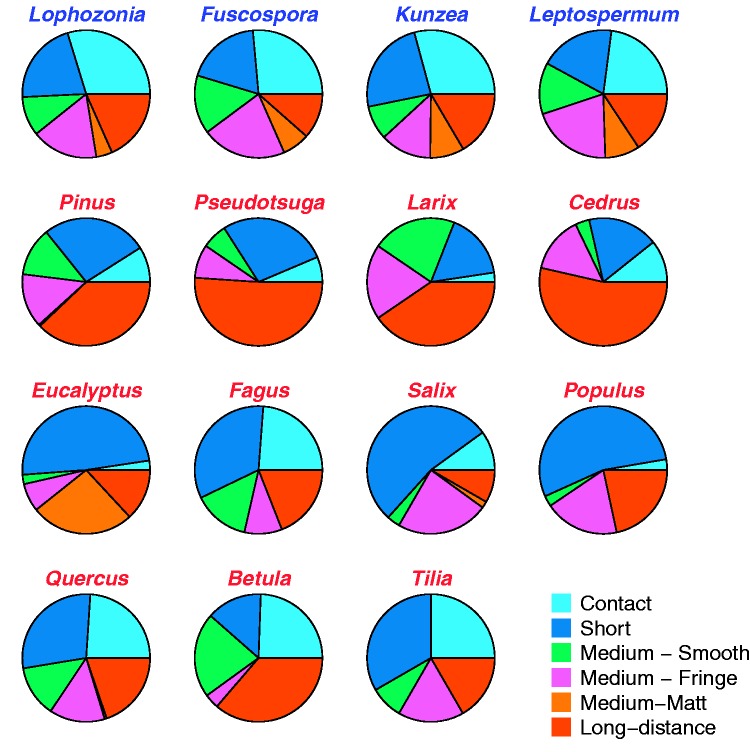
Functional composition of ectomycorrhizal fungal exploration types on native and alien tree genera in New Zealand by tree genus. Native genera names labelled in blue, aliens in red. Four alien tree genera with fewer than eight observations were excluded from functional analysis.

**Figure 2:**
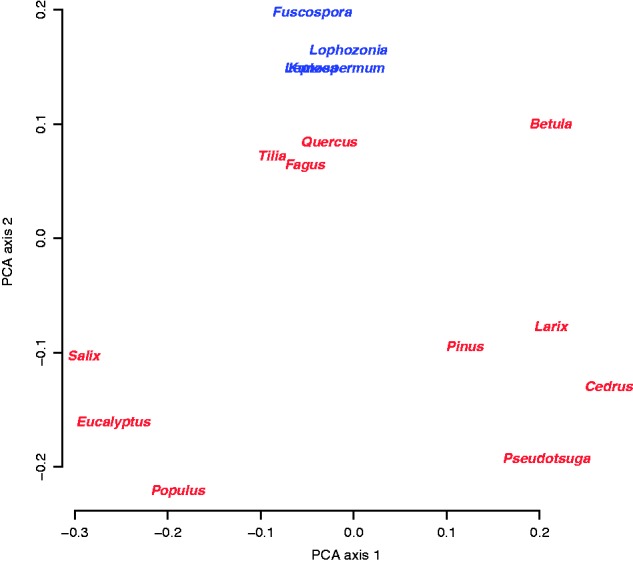
Multivariate visualization of functional similarity of fungal communities on native (blue) and alien (red) tree genera in New Zealand, based on data presented in Figure 1. The native genera *Kunzea* and *Leptospermum* overlap. Native tree genera had functionally similar communities of associated fungi, while alien tree genera had widely variable functional composition.

The four New Zealand native ectomycorrhizal tree genera had very similar mixtures of the six fungal exploration types (Fig. 1), resulting in very tight clustering in ordination (Fig. 2). Alien tree genera in New Zealand, in contrast, had very variable functional composition of associated fungal exploration types (Fig. 1), resulting in a greater spread in ordination (Fig. 2) and a highly significant difference in dispersion (betadisper, *F*_1,13__* *_ = _* *_15.7, *P  *=  0.0016). Native tree genera had all six functional types represented, with roughly equal proportions of contact, short-distance, medium smooth and medium-fringe exploration types; a smaller proportion of long-distance types, and the smallest representation of medium-matt types. Medium-matt types were formed by 13 different fungal genera (most common including *Hysterangium*, *Phellodon*, *Gallacea*, *Ramaria* and *Clavaria*) on native trees as well as on Australian *Eucalyptus*, but this functional type was largely absent from alien tree genera originating from the northern hemisphere.

The presence of all six functional types in relatively even distributions resulted in a higher Shannon diversity of functional types associated with native tree genera (*H*′  =  1.70) than alien tree genera (*H*′=1.39; *t*_12.83_ = *−*7.05, *P* < 0.001), which generally lacked the medium-matt forming type and tended to have a less even distribution of types (Fig. 1). Randomized simulations showed that the difference in *H*′ partially reflected a bias due to smaller sample size in alien species (randomized data *H*′  =  1.69 for natives and 1.55 for aliens), but the observed difference in native versus alien *H*′ values was larger than the difference in randomized simulations in >99.99 % of simulations.

**Figure 3:**
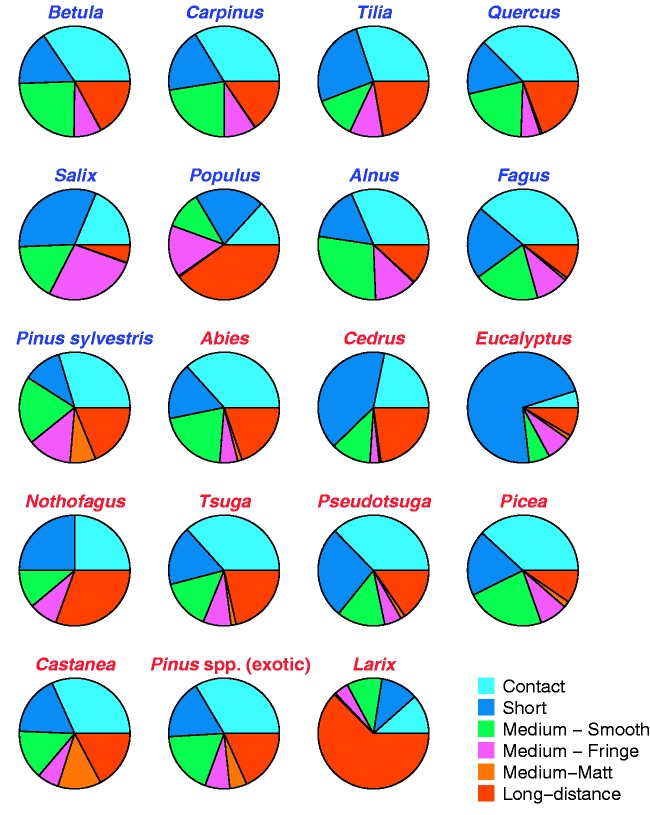
Functional composition of ectomycorrhizal fungal exploration types on native and alien tree genera in the UK by tree genus. Native genera names labelled in blue, aliens in red.

**Figure 4:**
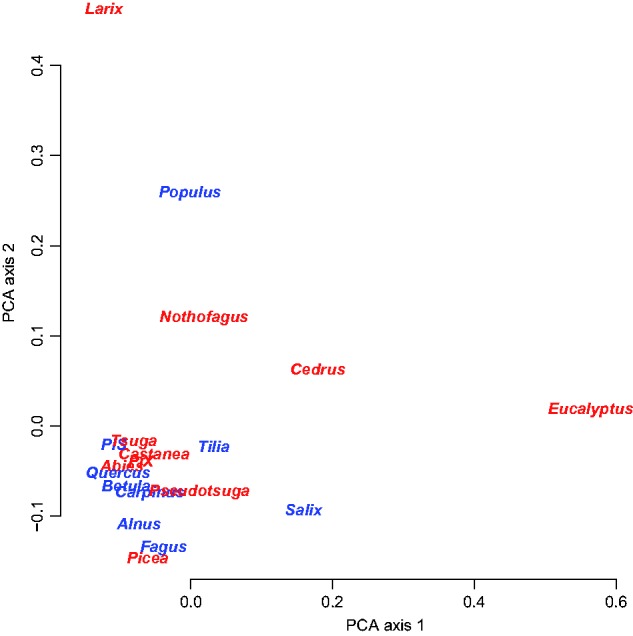
Multivariate visualization of functional similarity of fungal communities on native (blue) and alien (red) tree genera in the UK based on data presented in Figure 2.

The UK data showed strong functional similarity of hyphal exploration types across all tree genera, regardless of native versus alien status (Figs 3 and 4). Functional diversity was also not significantly different between native trees (*H*′  =  1.27) and aliens (*H*′  =  1.32; *t*_6.6 _ = _ _0.21, *P*  =  0.84) and there was no significant difference in dispersion (betadisper, *F*_1,17 _=0.92, *P * =  0.35).

### Network structure and modularity

The New Zealand bipartite interaction network was significantly antinested (weighted nestedness  =  0.74 compared with 95 % confidence interval of random expectation of 0.77–0.81, *P* < 0.01). The UK bipartite interaction network followed a similar pattern (observed nestedness 0.71 versus random expectation 0.80–0.82; *P *< 0.01).

**Figure 5:**
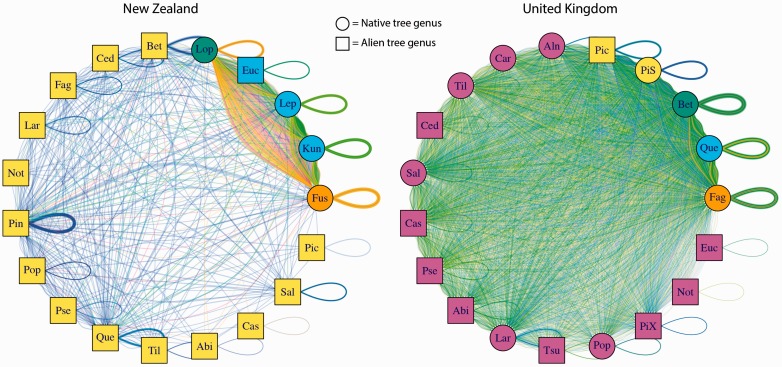
Interaction network of tree genera and ectomycorrhizal fungi in New Zealand (left) and the UK (right), showing plant–plant modules by the colour of the vertex. Native plant genera are indicated by circles, alien plants by squares, labelled with the first three letters of the genus except in the UK network, where PiS = *Pinus sylvestris*, and PiX = alien *Pinus* spp. Each link represents a fungal species either linking plant species or forming a self–self link (loops), with link width proportional to the square root of the number of observations (scaled 1/10th in the UK network compared with NZ network). The colour of the links indicates fungal-fungal modules (not discussed).

The NZ unipartite projected plant network clustered into 4 modules (modularity  =  0.46). All alien trees in New Zealand, with the sole exception of *Eucalyptus*, were clustered into one module within the network (Fig. 5). The Myrtaceae, including *Kunzea* and *Leptospermum* (both native) and *Eucalyptus* (alien) all formed a second module. Native *Lophozonia* and *Fuscospora* were each placed into an individual module, while *Nothofagus* (alien, but in the same family) was placed into a module with all other alien trees.

A similar pattern occurred in the UK, where there were five modules detected (modularity 0.38). The native tree genera *Fagus*, *Quercus* and *Betula* were each placed into an individual module, while native *Pinus sylvestris* formed a module with alien *Picea*. All other alien tree genera were placed in one large module along with six native tree genera (Fig. 5).

## Discussion

Ectomycorrhizal sporocarp collections from New Zealand and the UK provide some of the largest databases of plant–fungal interactions ever assembled. Prior studies of alien plant—ectomycorrhizal fungal associations have relied on detailed molecular identification of fungi on plant roots, which provides reliable data yet is generally limited by logistic constraints to small scale studies (Nuñez and Dickie, 2014). As such, most studies have only examined a very limited number of tree species at a time in relatively restricted portions of their distributions (Dickie *et al.* 2010; Bogar *et al.* 2015; Wood *et al.* 2015; Nuñez *et al.* 2009; Hayward *et al.* 2015*a*, *b*; Moeller *et al.* 2015). Using sporocarp observations, in contrast, we were able to obtain data on 19 tree genera in each of two countries, allowing the first analyses of how functional types of mycorrhizal fungi differ between natives and aliens, and how alien tree—ectomycorrhizal fungal communities integrate into native interaction networks.

Our databases were reliant on sporocarp observations and the ability of observers to infer the host of a particular fungus. We acknowledge that sporocarp observations record only a fraction of the fungi associated with a plant species, and are unlikely to be strongly correlated with belowground abundance. Species that produce belowground fruiting bodies (truffles), cryptic and difficult to identify sporocarps (e.g. *Tomentella*), or are asexual are less likely to be recorded than species with highly visible sporocarps (e.g. *Amanita*, *Russula*). Furthermore, there may be biases among mycologists and citizen scientists in which fungi are collected and recorded. Nonetheless, there is no reason to expect these biases would be systematic and using sporocarp collections allowed us to obtain a large number of association records across a wide area of each country at minimal cost. Finally, we note that there is an extensive prior scientific literature using sporocarp observations for the study of alien fungi and their host associations (Chu-Chou and Grace 1982, 1990; Pringle and Vellinga 2006; Vellinga *et al.* 2009; Wolfe and Pringle 2012).

The remarkably consistent composition of functional types of fungi associated with native NZ trees, including two plant families, suggests a convergence on similar functional composition. Alien trees, in contrast, had a much more variable functional composition of associated fungi. The lack of a directional shift suggests this was not driven by ecosystem modification favouring one particular functional type. Instead, we suggest that limited co-introduction of fungi is creating variable communities of fungi on different alien trees. In some cases, this lack of functional diversity seems to have little consequence; *Pinus*, for example, can be a highly successful invader with as little as a single fungal symbiont (Hayward *et al.* 2015*b*). Nonetheless, functional diversity of symbionts is potentially important as a physiological determinant of plant growth, and may limit the ability of some alien trees to become invasive (Richardson *et al.* 2000; Diez 2005; Dickie *et al.* 2010). Trait-based analyses of alien plants (e.g. Van Kleunen *et al.* 2010) may need to consider the degree to which plant traits vary depending on available symbionts or might be modified by any subsequent acquisition of fungal symbionts.

The difference in functional diversity of native versus alien tree genera in New Zealand was not replicated in the UK dataset, and there was much less difference in fungal species diversity between alien and native plants in the UK (Table 1). This may reflect distance of transport. Most UK alien species were from elsewhere in the Northern hemisphere and therefore may be more phylogenetically ‘familiar’ to native fungi. This is supported by Bahram *et al.* (2013) who found many native fungi on alien *Pinus* in Iran, compared with the prevalence of alien fungi on *Pinus* in the Southern Hemisphere (Nuñez *et al.* 2009; Dickie *et al.* 2010; Walbert *et al.* 2010).

Alien trees in both archipelagos were mostly linked into large, non-specific modules via their fungal associations. In NZ this large module was exclusively dominated by alien trees, while in the UK it included both native and alien species. This is consistent with the concept of alien species behaving as generalists and hence reducing network modularity and increasing nestedness (Santos *et al.* 2012; Traveset *et al.* 2013). Supporting this, the fungi associated with native trees in both NZ and the UK had a lower host breadth (or, conversely greater host preference) than fungi associated with aliens. Increased network nestedness is expected to make interaction networks more stable. This has been widely recognized in ectomycorrhizal interactions, albeit not in a network theory context, as the role of generalist host plants in maintaining ectomycorrhizal fungal inoculum following disturbance (e.g. Perry *et al.* 1989; Hagerman *et al.* 2001).

Increased sharing of fungal species associations may be the outcome of multiple processes. Highly host-specific fungi may not have been co-introduced with plants, resulting in higher reliance on generalist fungi capable of forming novel associations with alien trees (Tedersoo *et al.* 2007; Bahram *et al.* 2013). Plant–fungal associations may also become less specific in the alien range. For example, Bogar *et al.* (2015) note a number of fungi shared between *Alnus* and *Salix* in New Zealand that would not typically be shared in their native range. Very host-generalist alien fungi may also form novel associations with alien plants from other origins when both are alien, such as *Amanita muscaria* from Europe associating with *Eucalyptus* in New Zealand (Nuñez and Dickie, 2014). Finally, both fungal and plant communities are highly responsive to soils and other habitat conditions. As noted by Zobel and Opik (2014), this can result in co-occurrence driven by habitat rather than symbiont specialization. The process of introduction may break this habitat linkage. In particular, a higher proportion of observations of fungi on alien trees are likely to be in plantations or of other planted trees, rather than soil types where a tree naturally occurs. Homogenization of fungal communities may further occur in commercial tree nurseries, through uniformity of soils used and through strong selection for fungi able to survive these conditions. Where alien trees become invasive, they may also occur on a wider range of soils than in the native range (plant edaphic niche expansion; Erfmeier 2013), reducing the influence of soil habitat as a driver of plant–fungal associations.

Shared symbionts are known to play an important role in natural succession, with pioneer vegetation facilitating the establishment of later successional plants (Horton *et al.* 1999; Dickie *et al.* 2004; Nara and Hogetsu 2004). Although our data cover alien plants in general, many alien ectomycorrhizal plants are also invasive (Richardson 1998; Rundel *et al.* 2014). In the context of invasion, shared networks of symbionts may allow early establishing plants to facilitate subsequent invasion. This is likely, for example, in the invasion of *Alnus* into areas previously invaded by *Salix* in New Zealand (Bogar *et al.* 2015), and possibly in the increased ectomycorrhization of *Pseudotsuga* in areas where *Pinus* had previously invaded (Dickie *et al.* 2014).

Some of the patterns we observe may reflect phylogeny, as there are only two phylogenetic pairs represented in our NZ native ectomycorrhizal tree data, two Nothofagaceae and two Myrtaceae. Having low phylogenetic diversity can be an issue in statistical analyses of ectomycorrhizal functioning (Koele *et al.* 2012). The presence of a phylogenetic signal driving some of our results is clear in the Myrtaceae in NZ, where alien *Eucalyptus* forms a module with native *Kunzea* and *Leptospermum*. The UK data also suggest some similarity of fungal functional communities within the Fagales, although the two Salicaceae and some of the Pinaceae are highly divergent in functional composition within plant family (Fig. 4).

## Conclusions

The use of sporocarp collection data linked to fungal functional traits shows that fungal associates of alien trees in NZ have a lower functional richness and more variable composition than native tree genera. Given that different fungal exploration types exploit different soil resources (Hobbie and Agerer 2010) reduced diversity of symbionts may limit plant nutrient uptake capabilities. Nonetheless, the lack of a similar pattern in the UK dataset indicates that this pattern may depend on either environmental context and/or time since introduction. Both datasets suggest that alien trees generally integrate into networks as large inter-connected modules, which contrast with the much smaller modules generally formed by native trees. Whether this is driven by fungal traits (e.g. lack of host-specific fungi; expanded host-range) or habitat drivers (e.g. planting into atypical soils) requires more detailed investigation to unravel. Regardless of the cause, the sharing of symbiotic fungi among alien trees may have important implications for the invasion process, if tree species which share fungal associates facilitate each other.

## Sources of Funding

I.A.D., P.E.H. and J.L.B. are funded by Tertiary Education Commission CORE funding to the Bio-Protection Research Centre. J.A.C. is funded by the New Zealand Ministry of Business, Innovation and Employment.

## Contributions by the Authors

I.A.D. conceived the study based partially on conversations with P.E.H. and J.B. J.C. had a major role in developing the fungal databases for NZ and the UK and assisted in obtaining, formatting, and interpreting fungal data. S.T.B. obtained similar data for North America, which helped inform the analysis but was not ultimately used in the final manuscript. I.A.D. conducted all analysis with input from J.L.B. and wrote the paper based on discussions with all authors. All authors helped improve the manuscript in revisions.

## Conflicts of Interest Statement

None declared.

## Supplementary Material

Supplementary Data

## Supporting Information

The following additional information is available in the online version of this article —

 **Table S1.** Ectomycorrhizal sporocarp and tree dataset for New Zealand.

 **Table S2.** Ectomycorrhizal sporocarp and tree dataset for UK.
